# Supplementary material for: The Evaluation of a Porcine Circovirus Type 2 (PCV2) Intradermal Vaccine Against a PCV2 Field Strain
Source: Vaccines (Basel). 2025 Mar 24;13(4):343. doi: 10.3390/vaccines13040343 (PMC12030864; doi:10.3390/vaccines13040343)
Supplement: Supplementary file 1 [file vaccines-13-00343-s001.zip › vaccines-3490834-supplementary.pdf]

Supplemental Table S1. Comparison of the area under curve of the PCV2 load.

Groups	Ear tags	Area Under the Curve	*P* value[a]
Control	605	19.43	0.003
	607	18.29	
	608	15.26	
	609	23.35	
	610	13.3	
	611	15.89	
	**Total**	**105.52**	
Vaccine	612	2.633	
	613	3.736	
	615	4.662	
	618	4.713	
	619	3.776	
	620	2.486	
	**Total**	**22.006**	

[a] Statistical analysis of the area under the curve of the PCV2 viremia between two groups. $P < 0.05$ indicated a statistically significant difference between the control and vaccine groups.
